# Linear discriminant analysis based predator-prey analysis of hot electron effects on the *X*-pinch plasma produced *K*-shell Aluminum spectra

**DOI:** 10.1038/s41598-019-47997-6

**Published:** 2019-08-14

**Authors:** Mehmet Fatih Yilmaz, Yusuf Danisman, Jean Larour, Leonid Arantchouk

**Affiliations:** 1 Basic Sciences, Engineering Department, Imam Abdulrahman Bin Faisal University, Dammam, Saudi Arabia; 2 Department of Mathematics and Computer Science, Queensborough Community College CUNY, New York City, NY USA; 3 Laboratoire de Physique des Plasmas (LPP), Ecole Polytechnique, UPMC, CNRS, U-PSud, OBSPM, Palaiseau, France; 4 Laboratoire d’Optique Appliquée, ENSTA ParisTech, CNRS, Ecole Polytechnique, Université Paris Saclay, Palaiseau cedex, France

**Keywords:** Statistics, Magnetically confined plasmas

## Abstract

In this study, Linear Discriminant Analysis (*LDA*) is applied to investigate the electron beam effects on the *X*-pinch produced *K*-shell Aluminum plasma. The radiating plasma is produced by the explosion of two 25-μm Al wires on a compact *L-C* (40 kV, 200 kA and 200 ns) generator, and the time integrated spectra are recorded using de Broglie spectrographs. The ion and electron oscillations of K-shell Al plasma are extracted using *LDA* of spectral database of non-LTE K-shell Al model. A three dimensional representation of *LDA* shows that the presence of electron beam exhibits outward spirals of Langmuir turbulence and the center region of the spirals recieves lower electron temperatures of 50–100 eV. These spirals then are modeled by logistic growth of predator-prey model. This modeling suggests that the ions (LD1: most dominant eigenvector of LDA) and electrons (LD2: second most dominant eigenvector of LDA) represent the predators and preys, respectively. Besides, addition of electron beams transforms evanescent oscillations to the standing ones.

## Introduction

Suprathermal electrons in plasmas is a trending topic of inertial confinement fusion and high energy density physics. Hot electrons are diagnosed with many different experimental and computational techniqes, such as *x*-ray emission, electron breamsstrahlung and *K*α emission, spectropolarimetry and particle-in-cell^1–4^. Collisional radiative models with non-Maxwellian electron distribution is another alternative method to diagnose hot electrons in an emission spectra. Abdallah *et al*. has compared calculated and experimental emission spectra of laser produced *K*-shell Al plasma. The calculations have been carried out using an electron energy distribution which includes both thermal and hot electron components being the parts of a detailed collisional-radiative model. The comparison of spectra has shown that, the presence of hot electrons can alter the spectroscopic interpretation of electron density derived from standard thermal methods^5^.

*X*-pinch produced plasmas are an alternative and unique source of hot electrons. Due to the existence of a strong electric field oriented along the axis of the interelectrode gap, it is expected that hot electrons affect collisional and radiative process in these plasmas. Moreover, the very fast timescale with subnanosecond *x*-ray bursts coming from hot spots urges to use models far from the local thermodynamic equilibrium (*LTE*). Hansen *et al*. studied in detail that collisional excitation and ionization rates of *K*- and *L*-shell non-*LTE* collisional-radiative atomic kinetics models are highly sensitive to the fraction of hot electrons^6^.

Plasma electron temperature and density are diagnosed using the ratio of resonant to resonant or resonant to intercombination of emission lines. This procedure becomes challenging in the presence of hot electrons which can affect the individual line ratio changes in the spectra. Modeling of such plasmas requires hybrid electron distribution function in collisonal radiative models which have maxwelian and nonmaxwelian portions. So, the fraction of hot electrons, half-width half-maximum of electron beam energy standing beside plasma electron temperature, increases the dimension of the database and hence the prosecution time. Since pattern recognition techniques such as principal component analysis (*PCA*) and linear discriminant analysis (*LDA*) can reduce the dimension of a dataset while preserving its originality, they are extensively used in spectroscopy of astrophysical plasmas^7–9^.

Origin of the hot electrons generation, involved in the Langmuir turbulence, is another challenging work in high energy density plasmas^10^. There are many studies conducted on the complex relation between suprathermal electrons and Langmuir turbulence^11^. Recently, it has been shown that the interaction between electron beams, and non-linear oscillations and microturbulence can be characterized by means of predator-prey models^12–15^.

In this work, we have applied linear discriminant analysis (*LDA*) method to investigate the electron beam effects on the spectra radiated by *X-*pinch produced *K*-shell Al plasmas^16^. The *LDA* coefficients obtained are modelled by logistic growth with predator (predator-prey models) to investigate the relation between electron beams, ions and microturbulence^17^. In^16^, *PCA* was used to perform data analysis whereas *LDA* has been used to explore the hidden structures of the spectra in this paper. The main difference between *PCA* and *LDA* is that *PCA* considers the data as a whole, however *LDA* considers the differences between the classes within the data. Therefore, *LDA* is expected to provide more information about the details of the hidden structures. For example, it is shown that *LDA* extracts information about the plasma (ion and electron) oscillations and turbulence nature while *PCA* exracts the temperature diagnostics and collective behaivor of the plasma in the presence of the electron beams^16^. By reducing the dimension, spectra can be visualized in three dimension. Besides, since the eigenvectors of *LDA* considered in this paper are the dominant ones, they carry important information about the spectra.

The paper is organized as follows. After describing briefly the experiment in Sec. II, the third section provides the details of the non-*LTE* collisional radiative model and studies the electron beam effects on the *K*-shell Al calculated spectra applying linear discriminant analysis and predator-prey dynamics. Sec. IV discusses the modelling of experimental data, and conclusive remarks are given in Sec. V.

## Experiment

**Figure 1:**
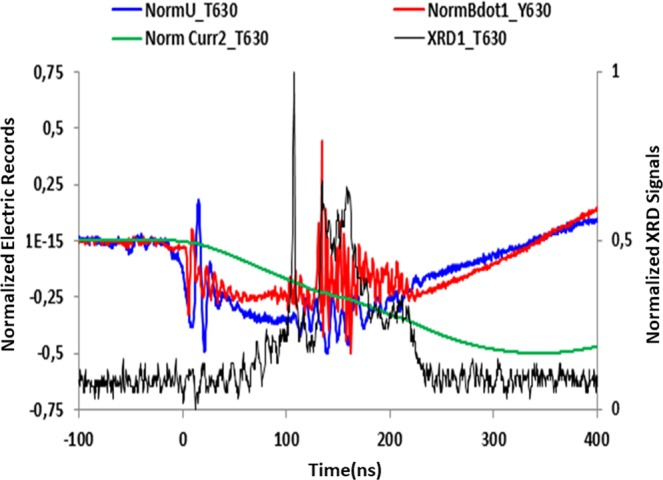
Close-up of the electrical and photonic records 500 ns around the time of pinching (shot *XP*_630). The electrical records (voltage, *B*-dot probe signal, current as numerically integrated from *B*-dot) are normalized (−1 to 1) to their span over the whole shot (left scale). *X*-ray signal is figured out by the *XRD* signal in volt (right scale), normalized (0 to 1). Close after the *x*-ray emission, a rise of the inductance leads to a current dip.

The mounting is a classical one and it has been described previously^16^. A compact pulsed power generator is devoted to the creation of point-like *x*-ray sources in the keV range for radiographical application to low contrast objects. The plasma is produced by the explosion of two 25-μm Al wires on a compact *L-C* (40 kV, 200 kA, 200 ns) generator and the denser and brighter spots sit close to the crossing point, thus the device ensures creation of a localized and reproducible plasma. The time integrated spectrum is recorded on *x*-ray film through de Broglie *KAP, PET* or mica spectrographs installed in the equatorial plane. Due to the limited number of photons in the *K*-shell range, a rather wide (5 mm) entrance slit was used and it was not possible to get any spatial resolution along the *X*-pinch axis. Simultaneously, a set of fast detectors (filtered, absolute *XUV p-i-n* diodes and photoconductive diamonds) were recording the time dependence of *x*-ray flux in the keV region and in ns- and sub-ns regime (see Fig. 1). Time-integrated pinhole imaging in the same spectral region was performed radially on *DEF x*-ray film. The last two records were ensuring afterwards that there was a unique small-extension bright spot or, at least, a much brighter one, ensuring that there was no overlay of spectra coming from different sources nor geometrical blurring.

**Figure 2:**
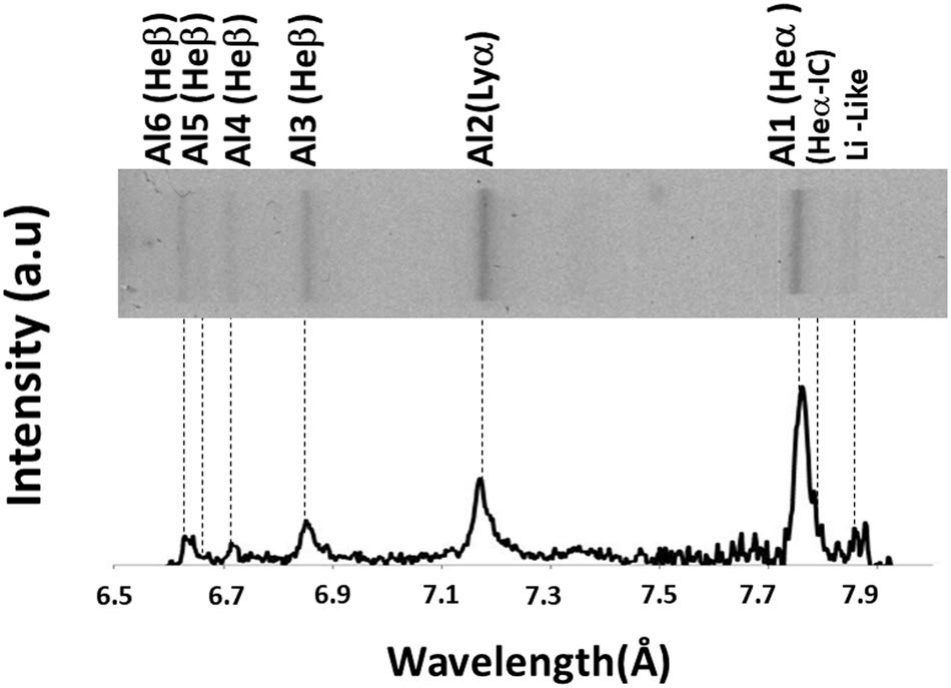
Time integrated spectrum of *K*-shell Al plasma (shot XP_630) with diagnostically significant lines.

Over many tests with various wires, showing evidences of *K*-shell Al, *L*-shell Cu and Mo, the present work deals with the electron temperature and density and hot electron beam fractions of *X*-pinch produced *K*-shell Al plasma. Figure 2 presents a time-integrated *x*-ray spectrum of *K*-shell Al plasma (shot XP_630). The spectral lines of the resonant transitions of Al1 (He-like), Al2 (H-like), Al3 (He-like) and Li-like Al as well as satellite transitions of Al1, Al2 and Al3 are well resolved^16^.

## Electron Beam Effects on the Synthetic Spectra of *K*-Shell Aluminum

### Non-LTE collisional radiative model of *K*-Shell Al

The details of non-LTE *K*-shell Al calculated spectra using collisional radiative model and the principal component analysis have been described previously^16^. In brief, the energy levels, collisional and radiative rates and cross-sections’ calculations were performed using the Flexible Atomic Code (FAC)^18^. Collisional radiative model uses the hybrid electron distribution function *F(e)* = *(1* − f)*F_*maxwellian*_ + f*F_*non-maxwellian*_ to charecterize the effects of hot electrons on the spectra. Gaussian distribution with the centered energy of *E*_0_ = 10 keV was used to describe the fraction of the hot electrons. Voigt profiles of line broadening with the resolution of 300 have been used for fitting the broadening of the experimental spectra^16^.

### Linear Discriminant Analysis

The effects of electron beams in the emission spectra can be diagnosed by the ratio of the lines which are sensitive to the high energy electron population, such as the ratio of different ion charge states of (Al1 + Al1 − IC)/Al2 and same ion charge states of (Al1 + Al1 − IC)/Al3^16^.

Pattern recognition techniques are also alternative approaches to diagnose plasmas. Principal Component Analysis (*PCA*) and Linear Discriminant Analysis (*LDA*) are the most commonly used techniques for the feature extraction and classification of high dimensional data. Recently, *PCA* has been applied to diagnose electron beam effects in *X*-pinch produced *K*-shell Al plasmas and the details of the extracted principal components over *K*-shell Al model can be found elsewhere^16^. However, the brief explanation of *PCA*, *LDA* and unified version of *PCA* and *LDA* are given as below.

*PCA* is an unsupervised method which reduces the dimension of the data but preserves its characteristics. In *PCA*, the vectors which have the largest variance associated to the data are computed and used as a basis for the new reduced space. Therefore, each original data is represented by its coordinate vector in the reduced space with a lower dimension.

Let *Γ* be a NxM matrix whose cloumns consist of the original dataset. Let *μ* be the mean value of the columns of *Γ*, and *Φ* be the matrix obtained from *Γ* by subtracting *μ* from the each column of *Γ*. The covariance matrix, which is a measure of how much variables change together, is $\frac{1}{M}\mathop{\sum }\limits_{i=1}^{M}\,{{\Phi }}_{i}{{\Phi }}_{i}^{t}$ where ${{\Phi }}_{i}$ is the *i-*th column of *Φ* and superscript *t* means transposed. The vectors which has the largest variance are the eigenvectors of covariance matrix with largest eigenvalues, called principal components and denoted by |PC1>, |PC2>, |PC3>, … depending on the order of the corresponding eigenvalues.

In applications, the vector space generated by the principal compenents that correspond to the most dominant eigenvalues are considered in order to reduce the dimension.

For instance, let |PC1>, |PC2>, |PC3> be the principal components which corresponds to the largest three eigenvalues. If *v* is any vector of ${{\mathbb{R}}}^{{\rm{N}}}$ then the vector $v$ can be represented by three coordinates, and in the new three dimensional vector space the coordinate vector of *v* is (*v*●|PC1>, *v*●|PC2>, *v*●|PC3>), where ● is the Euclidian dot product.

On contrary, *LDA* is a supervised method which also reduces the dimension of the dataset. In *PCA*, data is considered in its entirety whereas in *LDA* the focus is on the characteristics of the different classes to discriminate them. Let a dataset consists of *K* classes of *N* × 1 vectors, where each class contains *M* vectors. Let *Γ*_*i*_^*j*^ be the i’th element of the class *j* for *i* = 1, 2, …, *M* and *j* = 1, 2, …, *K*. Let *μ*_*j*_ be the mean of the class *j*, and *μ* be the mean of all classes. Then the within-class scatter matrix *S*_*w*_ and the between-class scatter matrix *S*_*b*_ can respectively be expressed as:

1 $${S}_{w}=\frac{1}{K}{\Sigma }_{j=1}^{K}{\Sigma }_{i=1}^{M}\,({{\Gamma }}_{i}^{j}-{\mu }_{j})\,{({{\Gamma }}_{i}^{j}-{\mu }_{j})}^{t}$$

2 $${S}_{b}={\Sigma }_{j=1}^{K}\,({\mu }_{j}-\mu ){({\mu }_{j}-\mu )}^{t}.$$

In *LDA*, the eigenvectors of *(S*_*w*_)^*−1*^. *S*_*b*_ which correspond to largest three eigenvalues are considered, and they are denoted by |LD1>, |LD2> and |LD3>. In *LDA*, there is a difficulty in taking the inverse of the matrix *S*_*w*_ if it is large. Therefore, *LDA* has a disadvantage in processing high dimensional data. To find a remedy for this problem in applications, the dimension of the original data is first reduced by *PCA* before appying *LDA* algorithm. In this paper, a unified *PCA* and *LDA* algorithm is applied to the spectra clustering^19–22^.

### Spectral Representation of *LD* vectors

In the present work, as the first step, *PCA* is used to reduce the size of *S*_*w*_ for each electron beam fraction so that it can be inverted. The data set of any fraction consists of 230 = 5 (densities: 1 × 10^19^, 5 × 10^19^, 1 × 10^20^, 5 × 10^20^ and 1 × 10^21^ cm^−3^) × 46 (temperatures: 50, 60, …, 500) spectra of size 456 × 1. By applying *PCA*, the dimension is reduced to 40 by means of projecting each of the spectra into the space generated by the most dominant 40 PCs. As the second step, *LDA* is applied to the 5 classes of fractions where each class consists of 230 vector of size 40 × 1, and the dimension is reduced to 3. The new vector space is generated by |LD1>, |LD2> and |LD3>.

**Figure 3:**
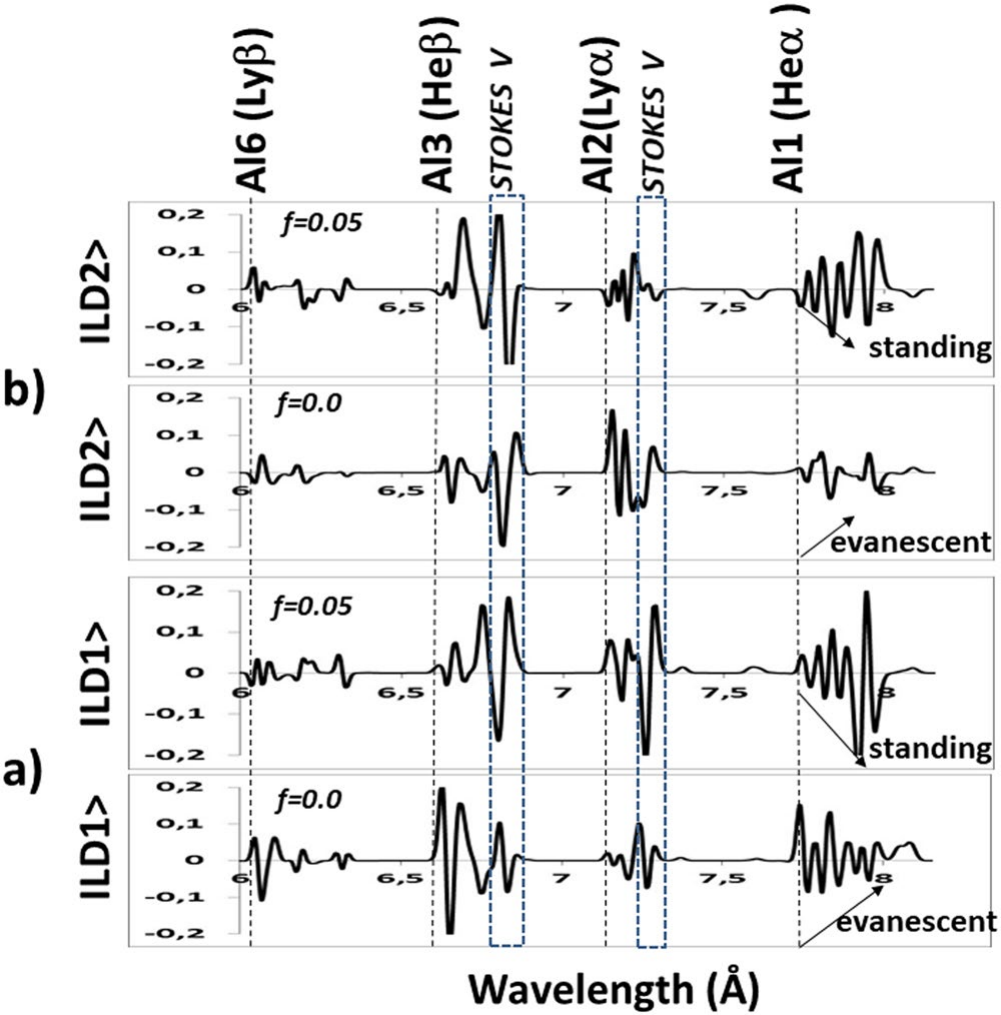
Main features of the (**a**) ion (|LD1>) and (**b**) electron (|LD1>) waves with electron beam fractions *f* = 0.00 (none) and *f* = 0.05.

In Fig. 3, the vector spectra of ions (|LD1>) and electrons (|LD2>) are given for the beam fraction ranging from *f* = 0.00 to f = 0.20. Figure 3 a and b show that, the resonant transitions of Al1, Al2, Al3, Al4, Al5 and Al6 are almost absent, but their oscillations are present. Addition of the electron beams transform the evanescent waves to the standing waves^23^. By comparing the response of resonant transitions to the electron beams in *PCA* vector spectra in our recent works^16^, we have shown that *LDA* can clearly discriminate the behavior of oscillations of ions and electrons in the presence of electron beams^16^.

### Three-Dimensional Representation of LDA and predator-prey dynamics

**Figure 4:**
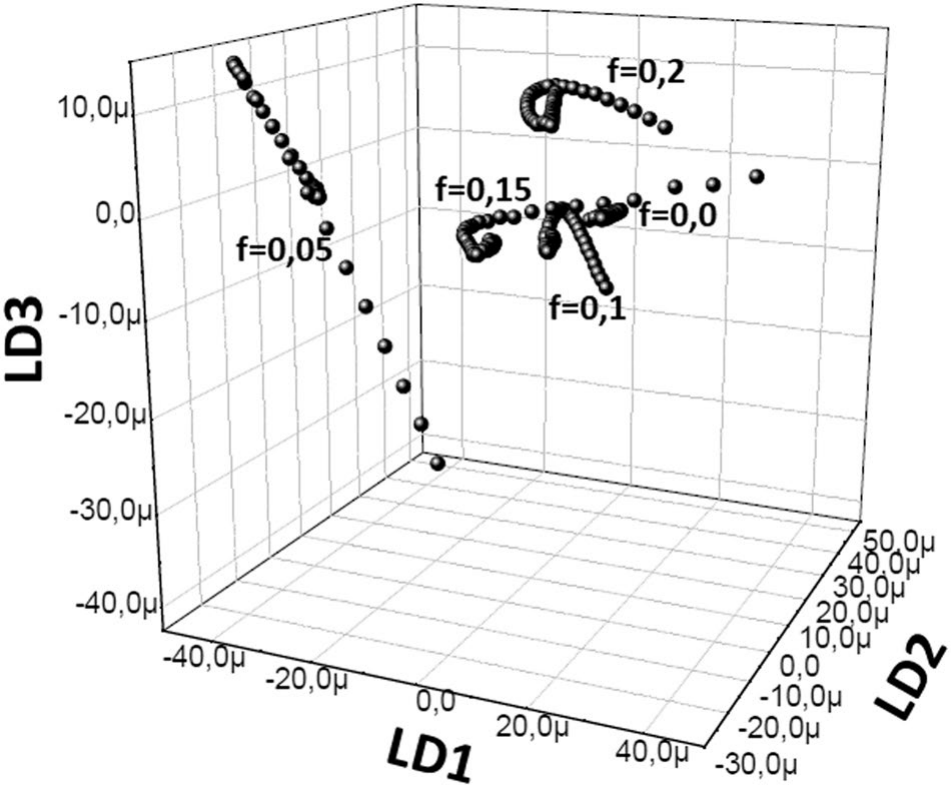
(|LD1>, |LD2>, |LD3>) points for the electron beam fractions between $f=0.00$ and $f=0.20$ at the electron density of *n*_*e*_ = 1 × 10^20^ cm^−3^. Th_*e*_ µ value has been introduced in Sec.III.b.

Figure 4 shows the behavior of |LD1>, |LD2> and |LD3> coefficients with temperature increase for the fraction groups *f* = 0.00, 0.05, 0.10, 0.15 and 0.20. The fraction case 0.2 especially shows that the temperature increase results in plasma oscillations to move in an outward spiral turbulence.

#### Predator-Prey model

Gurcan *et al*. stated that the turbulence in hot dense plasma can be characterized by the predator-prey of population models^14,15^. The populations of two species interacting as a predator and prey can be modeled by using a pair of nonlinear, first-order equations which are the modified versions of the original Lotka–Volterra equations^24^. These models are called Predator-Prey models and they have been applied to many different areas such as chemical reactions^25^, astronomy^26^, economics^27–30^, plasmas^13,15,22^, and evolutionary game theory^26^ in order to express the complicated real life situations as differential equations.

The Lotka–Volterra equations^24^ are two non-linear and first-order differential equations:

3 $$\frac{dP}{dt}=[r-sQ]P\,{\rm{and}}\,\frac{dQ}{dt}=(\,-\,u+vP)Q$$

where $P$ and $Q$ are populations of prey and predator, respectively. $r,\,s,\,u,\,v$ are parameters describing the interaction of the two species. Originally, these equations were used to exhibit the relation in a biological system of two interacting species: predator (Q) and prey (P).

**Figure 5:**
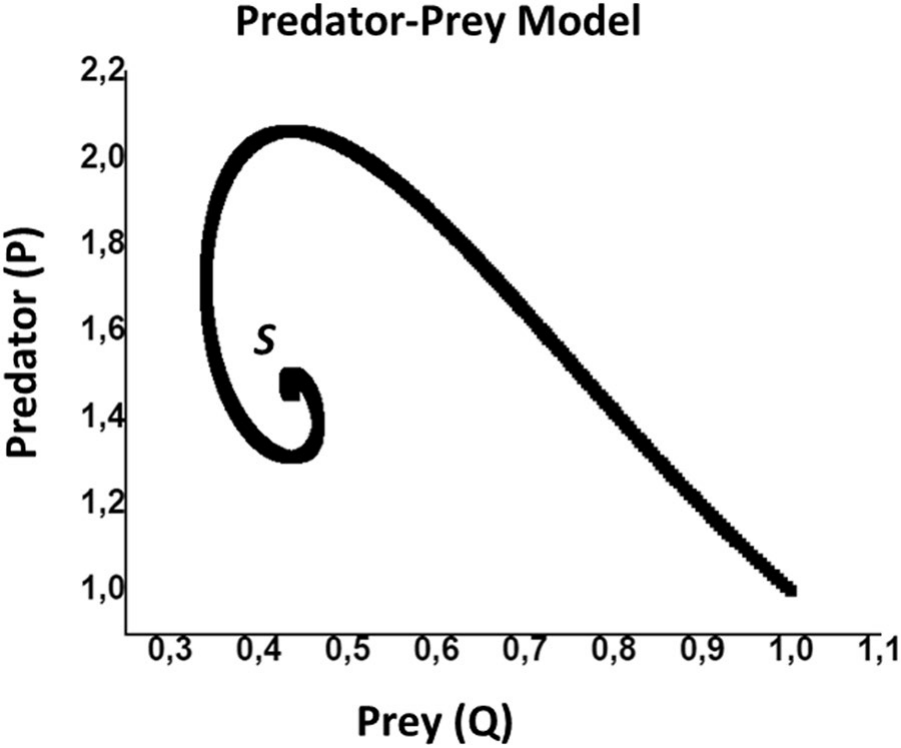
Scheme of a system trajectory in Predator-Prey model.

Introducing a constraint on carrying capacity of the prey population forces to modify the original Lotka–Volterra equations and yields^31^:

4 $$\frac{dP}{dt}=[r(1-\frac{P}{K})-sQ]P\,{\rm{and}}\,\frac{dQ}{dt}=(-u+vP)Q$$

where $K\,\,$is the maximum size of preys. This is modified Lotka-Volterra model with capacity constraint. For a particular choice of parameters, the graph of P versus Q is given in Fig. 5.

There is a steady state at the point $\,S=[\frac{u}{v},\frac{r}{s}(1-\frac{u}{vK})]$ and trajectory spirals around the steady state point *S*. There are two cases, either trajectories will spiral inwards and points converge to *S* or they spiral outwards and the points diverge from *S*.

In our case, Predator-Prey model is used to exhibit the characteristics of the plasma via the coefficients of linear discriminant analysis in Fig. 3. As a result of linear discriminant analysis for *f* = 0.2, it is shown that |LD2> coefficients behave like prey and |LD1> coefficients behave like a predator as in Fig. 2. This is because |LD1> coefficients deplete the |LD2> coefficients. As it is known that radiative and dielectronic recombination transitions associated to the resonant transtions are due to electron capturing process, |LD2> and |LD1> coefficients represent the ions and electrons, respectively^13^. Since the trajectory spiral outwards, the steady state point *S* is unstable. On contrary, the trajectory spiral inwards and the central point is stable in the case *f* = 0.0.

**Figure 6:**
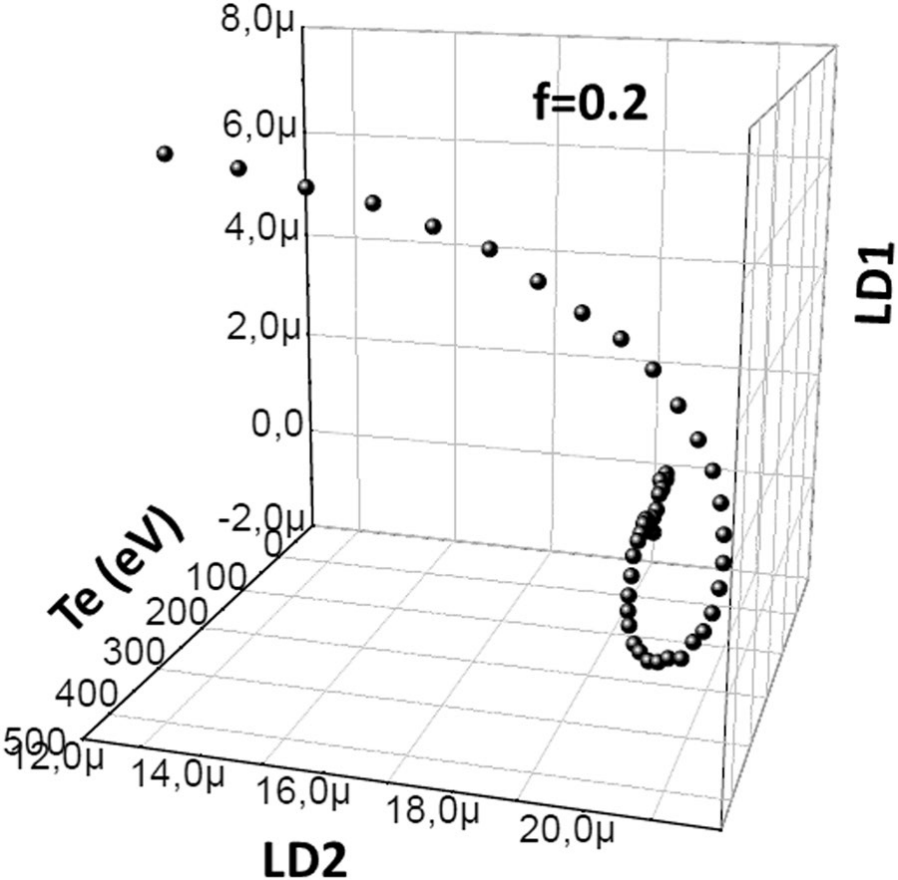
|LD1>and |LD2> coefficient *vs* plasma electron temperature.

An important result of our work is that the point *S* corresponds to the lowest temperature, and the center region of the spiral has lower temperatures (50–100 eV). On the other hand, on these low temperatures, the low ionization is fixed by the electron beams^13^. Therefore, as the temperature decreases (|LD2> coefficient, |LD1> coefficient) which points converge to *S* which is an accumulation point. The temperature path is given in Fig. 6 and, |LD1> and |LD2> coefficients have inverse correlation as temperature decreases to 300 eV. |LD1> and |LD2> coefficients are accumulated above this temprature.

Another key finding is that, the trend of the |LD1> and |LD2> coefficients can be predicted by using the Predator-Prey model. This estimation is more accurate than the ones in^16,32^ because in these papers only the information obtained by |PC1> is used whereas in this work |LD1> and |LD2> are used together for characterization, which means that less information is lost.

Since the Predator-Prey model that we are using has a carrying capacity for the |LD2> coefficients, we are also able to find a boundary for the |LD2> coefficients of the plasma.

## Modelling Using *PCA*, *LDA* and Non-*LTE* Models

**Figure 7:**
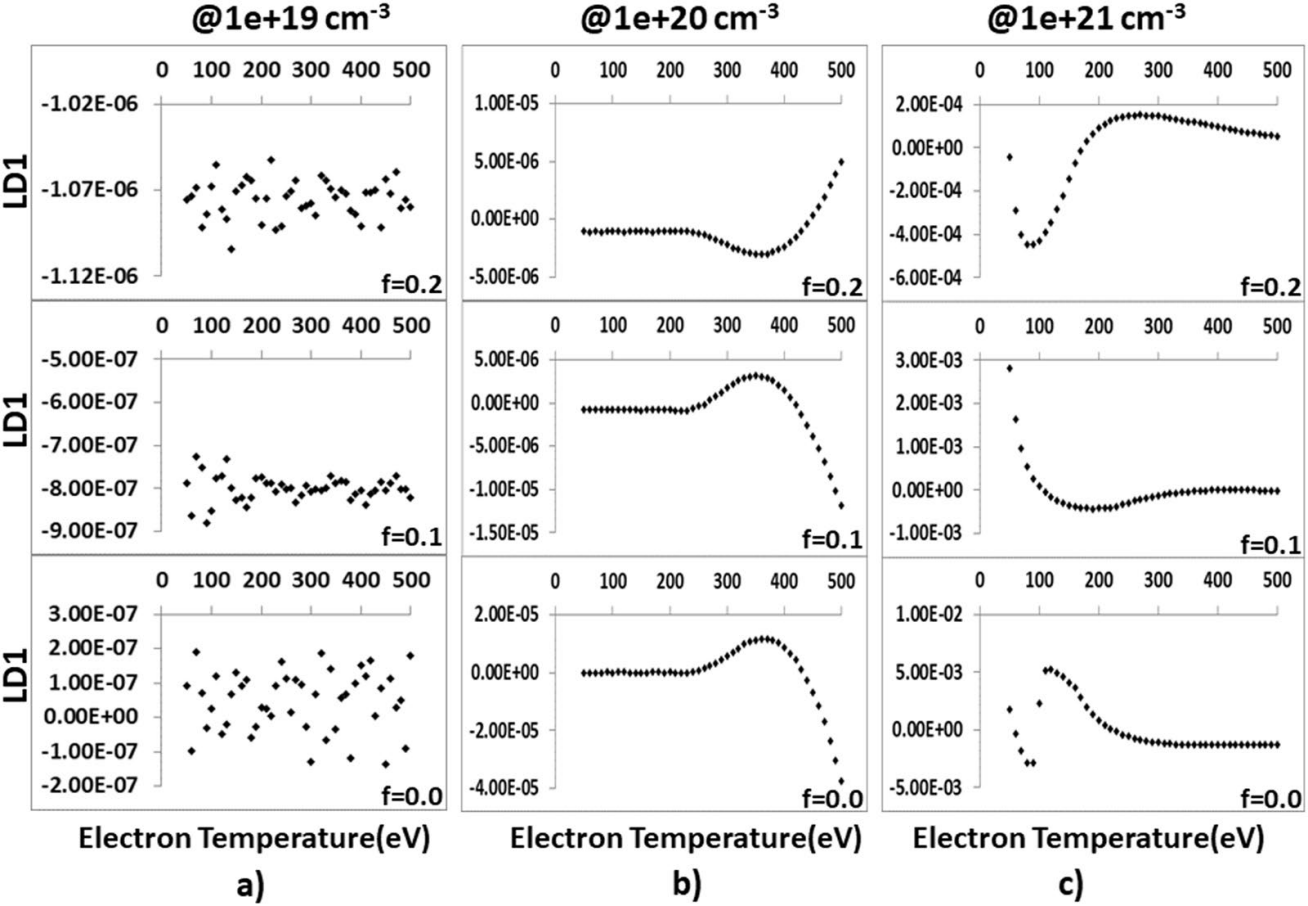
|LD1> coefficients versus electron temperatures graphs for certain electron densities and beam fractions.

In Fig. 7, for the electron densities of *n*_*e* = _1 × 10^19^, 1 × 10^20^ and 1 × 10^21^ cm^−3^, the |LD1> coefficients of the 46 × 3 spectra in the temperature range between 50 and 500 eV are given for the beam fractions of *f* = 0.00, 0.01 and 0.20. Figure 7 shows that |LD1> coefficients are more stabilized at *f* = 0.1 with the plasma electron density of 1 × 10^19^ cm^−3^. For the electron density of 1 × 10^20^ cm^−3^, |LD1> coefficients follow stabilized motion for the electron temperatures below 250 eV whereas they follow non-linear motion above that temperature. For the electron density of 1 × 10^21^ cm^−3^, |LD1> coefficients follow a nonlinear profiles for the electron temperatures below 250 eV, and they become stabilized above this temperature.

There is no resemblance to both of (Al1 + Al1 − IC)/Al2 and (Al1 + Al1 − IC)/Al3 ratios^16^ compared to the traditional ratio diagnostics of *K*-shell Al spectra. This result is expected because *LDA* focuses on the behavior of the oscillations rather than the strong characters of resonant transitions in *PCA* modeling^11^.

**Figure 8:**
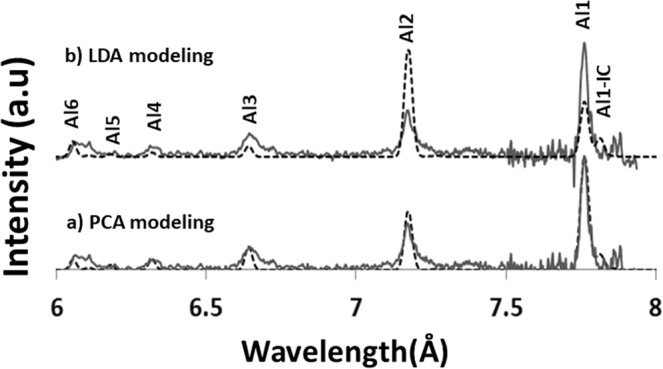
Experimental spectrum versus *LDA* and *PCA* model produced calculated spectrum (*T*_*e*_ = 100 eV, *n*_*e*_ = 5 × 10^20^ cm^−3^ and *f* = 0.2).

The modeling of the electron temperature of K-shell Al spectrum has been done first using PCA regression modeling^16^. This modeling gives the plasma electron temperature of 100 eV, density of 5 × 10^20^ cm^−3^ and f = 0.20 (Fig. 8). Since PCA and line ratio diagnostics correlate well, such an agreement is expected. However, by modeling using LDA at 100 eV with density n_e_ = 5 × 10^20^ cm^−3^, f = 0.2 and following regression curve of Fig. 7b, resonant transitions of Al1 and Al2 are not estimated well. This is due to the fact that LDA mainly focuses on oscillations of ions and electrons (Fig. 3).

## Conclusion

The first result of our present work is that *LDA* can be used for the data classification of non-*LTE* collisional radiative *K*-shell Al model, and each spectrum can be characterized by the dominant *LD* coefficients. *LDA* can also be used as an alternative plasma diagnostic of *K*-shell Al spectra, especially for providing insights for ion and electron oscillations. However, *PCA* realizes a better correspondence with line ratio diagnostics for estimation of plasma parameters. Modelling of a representative *K*-shell Al spectrum by using *PC* coefficients gives *T*_*e*_ = 100 eV, *n*_*e*_ = 5 × 10^20^ cm^−3^ and *f* = 0.2 (with the beam energy centered at 10 keV). The *LDA* vector spectra show that the addition of the electron beam leads the oscillations of Al1, Al2 and Al3 to move as standing waves. The plot of electron temperature, |LD1>, |LD2> and |LD3> coefficients (at electron density of *n*_*e*_ = 1 × 10^20^ cm^−3^) clearly shows that electron beam addition on the spectral model generates quantized clusters in the vector space and perform the oscillations in an outward spiral like turbulence^18^. Modelling of these turbulence using Predator-Prey model suggests that the center of the spiral recieves lower electron temperatures in where ionization is fixed by the electron beams. Another result of Predator-Prey modeling is that ions and electrons behave as predator and prey, respectively.

## References

[1] Chen, H., McLean, H. S., Patel, P. K. & Wilks, S. C. Hot Electron Measurement and Modeling for Short-Pulse Laser Plasma Interactions. LLNL report UCRL-JC-155353, 3rd Int. Conf. on Inertial Fusion Science and Applications, Monterey, CA, 7–12 sept (2003).

[2] Renner O, Smid M, Batani D, Antonelli L (2016). Suprathermal electron production in laser-irradiated Cu targets characterized by combined methods of x-ray imaging and spectroscopy. Plasma Phys. Contr. Fusion..

[3] Meadowcroft AL, Edwards RD (2012). High-Energy Bremsstrahlung Diagnostics to Characterize Hot-Electron Production in Short-Pulse Laser-Plasma Experiments. IEEE Trans. on Plasma Science.

[4] Kemp AJ, Sentoku Y, Tabak M (2008). Hot-electron energy coupling in ultraintense laser-matter interaction. Phys. Rev. Lett..

[5] Abdallah J (1999). Hot electron effects on the satellite spectrum of laser-produced plasmas. J. Quant. Spectr. Rad. Transfer.

[6] Hansen SB, Shlyaptseva AS (2004). Effects of the Electron Energy Distribution Function on modeled x-ray spectra. Phys. Rev..

[7] Suzuki N (2006). Quasar Spectrum Classification with Principal Component. Analysis (PCA): Emission Lines in the Lyα Forest. The Astrophysical J. Suppl. Series.

[8] Wang L (2011). Principal component analysis of the Spitzer IRS spectra of ultraluminous infrared galaxies. Monthly Not. Royal Astron. Soc. MNRAS.

[9] Kobel P, Hirzberger J, Solanki SK, Gandorfer A, Zakharov V (2009). Discriminant analysis of solar bright points and faculae - I. Classification method and center-to-limb distribution. Astronomy & Astrophysics.

[10] Vu HX, DuBois DF, Russell DA, Myatt JF (2012). Hot-electron generation by “cavitating” Langmuir turbulence in the nonlinear stage of the two-plasmon–decay instability. Physics of Plasmas.

[11] Yoon PH, Ziebell LF, Gaelzer R, Lin RP, Wang L (2012). Langmuir turbulence and suprathermal electrons. Space Science Reviews.

[12] Sara M, Anderson J, Gürcan OD (2015). Predator-prey model for the self-organization of stochastic oscillators in dual populations. Physical Review E.

[13] Ross AE, McKenzie DR (2016). Predator-prey dynamics stabilised by nonlinearity explain oscillations in dust-forming plasmas. Scientific reports.

[14] Berionni V, Gurcan OD (2011). Predator prey oscillations in a simple cascade model of drift wave turbulence. Phys. Plasmas.

[15] Obayashi S, Gurcan OD, Diamond PH (2015). Direct identification of predator-prey dynamics in gyrokinetic simulations. Physics of Plasmas.

[16] Yilmaz MF, Danisman Y, Larour J, Aranchuk L (2015). Principal component analysis of electron beams generated in K-shell aluminum X-pinch plasma produced by a compact LC-generator. High Energy Density Physics.

[17] Brauer, F. & Castillo-Chavez, C. *Mathematical Models in Population Biology and Epidemiology*. (Springer, 2001).

[18] Gu MF (2008). The flexible atomic code. Can. J. Phys..

[19] Danisman Y, Yilmaz MF, Ozkaya A, Comlekciler I (2014). A comparison of eigenvalue methods for principal component analysis. Appl. and Comput. Math..

[20] Khan A, Farooq H (2011). Principal component analysis-linear discriminant analysis feature extractor for pattern recognition. Int. J. of Computer Science Issues.

[21] A. Eleyan, & H. Demirel. PCA and LDA based face recognition using feedforward neural network classifier, in Multimedia Content Representation, Classification and Security, Vol. 4105 of the series Lecture Notes in Computer Science, B. Gunsel et al. ed., (Springer Berlin Heidelberg, pp. 199–206 2006).

[22] Bottorff M, Ferland G, Baldwin J, Korista K (2000). Observational Constraints on the Internal Velocity Field of Quasar Emission-Line Clouds. The Astrophys. J..

[23] Hayashi N (1995). Excitation mechanism of standing waves produced by electron beam plasma instability. Physics of Plasmas.

[24] Brauer, F. & Castillo-Chavez, C. *Mathematical Models in Population Biology and Epidemiology* (Springer, 2001).

[25] Esposito LW (2012). A predator-prey model for moon-triggered clumping in Saturn’s rings. Icarus.

[26] Hofbauer, J. & Sigmund, K. *Evolutionary Games and Population Dynamics*. (Cambridge University Press, 1998).

[27] Goodwin, R. M. *A Growth Cycle, Socialism, Capitalism and Economic Growth*, Feinstein, C.H. (ed.), (Cambridge University Press, 1967).

[28] Desai M, Ormerod. P (1998). Richard Goodwin: A Short Appreciation. The Economic Journal.

[29] Samuelson P (1971). Generalized Predator-Prey Oscillations in Ecological and Economic Equilibrium. Proc. Natl. Acad. Sci. USA.

[30] Martinez J, Chequer N, Gonzalez J, Cordova T (2012). Alternative methodology for gold nanoparticles diameter characterization using PCA technique and UV-VIS spectrophotometry. Nanosci. Nanotechnol..

[31] Allman, E. S. & Rhodes, J. A. *Mathematical Models in Biology: An Introduction* (Cambridge University Press, 2004).

[32] Hering R (1990). Oscillations in Lotka-Volterra systems of chemical reactions. J. Math. Chem..

